# HBx-Induced NF-κB Signaling in Liver Cells Is Potentially Mediated by the Ternary Complex of HBx with p22-FLIP and NEMO

**DOI:** 10.1371/journal.pone.0057331

**Published:** 2013-03-04

**Authors:** Keo-Heun Lim, Hyo Sun Choi, Yong Kwang Park, Eun-Sook Park, Gu Choul Shin, Doo Hyun Kim, Sung Hyun Ahn, Kyun-Hwan Kim

**Affiliations:** 1 Department of Pharmacology, and Center for Cancer Research and Diagnostic Medicine, IBST, Konkuk University School of Medicine, Seoul, Republic of Korea; 2 Institute of Functional Genomics, Konkuk University, Seoul, Republic of Korea; Bambino Gesù Children’s Hospital, Italy

## Abstract

Sustained activation of NF-κB is one of the causative factors for various liver diseases, including liver inflammation and hepatocellular carcinoma (HCC). It has been known that activating the NF-κB signal by hepatitis B virus X protein (HBx) is implicated in the development of HCC. However, despite numerous studies on HBx-induced NF-κB activation, the detailed mechanisms still remain unsolved. Recently, p22-FLIP, a cleavage product of c-FLIP_L_, has been reported to induce NF-κB activation through interaction with the IκB kinase (IKK) complex in primary immune cells. Since our previous report on the interaction of HBx with c-FLIP_L_, we explored whether p22-FLIP is involved in the modulation of HBx function. First, we identified the expression of endogenous p22-FLIP in liver cells. NF-κB reporter assay and electrophoretic mobility shift assay (EMSA) revealed that the expression of p22-FLIP synergistically enhances HBx-induced NF-κB activation. Moreover, we found that HBx physically interacts with p22-FLIP and NEMO and potentially forms a ternary complex. Knock-down of c-FLIP leading to the downregulation of p22-FLIP showed that endogenous p22-FLIP is involved in HBx-induced NF-κB activation, and the formation of a ternary complex is necessary to activate NF-κB signaling. In conclusion, we showed a novel mechanism of HBx-induced NF-κB activation in which ternary complex formation is involved among HBx, p22-FLIP and NEMO. Our findings will extend the understanding of HBx-induced NF-κB activation and provide a new target for intervention in HBV-associated liver diseases and in the development of HCC.

## Introduction

The cellular anti-apoptotic protein c-FLIP is an inhibitor of apoptosis triggered by a death receptor-mediated signal [1]. A number of studies have shown that c-FLIP_L_ blocks procaspase 8 activation through recruitment of the death-inducing signaling complex (DISC) by interaction with its death effecter domain (DED). Recruitment of procaspase 8 to the DISC for auto-processing is disturbed by competition with c-FLIP_L_ because both proteins share the common DED domain in their N-terminal regions [1]–[4]. Until now, three isoforms of c-FLIP (c-FLIP_L_, c-FLIP_S_, c-FLIP_R_) and their cleavage products including p22-FLIP have been discovered at the protein level. All c-FLIP isoforms and p22-FLIP have two DED domains, which are essential for the inhibition of death receptor-induced apoptosis [1]–[6]. Among them, p22-FLIP is generated by cellular cleavage from c-FLIP_L_ or c-FLIP_S_ and has been shown to induce NF-κB activation through interaction with the IκB kinase (IKK) complex in immune cells [6].

It is well-established that transcription factor NF-κB regulates a variety of cell functions such as inflammation, regulation of the immune system, apoptosis, stress response, differentiation, cell proliferation, and especially cancer development [7]–[8]. Mis-regulation of NF-κB is one of the causative factors for tumorigenesis through improper induction of various cell growth related genes (over 200 genes) and aberrant crosstalk with various signaling pathways, which are related to cell growth and survival pathways such as Ras/MAPK cascades, Wnt/β-catenin signaling, and PI-3 kinase/Akt pathway [8]–[9]. Constitutive activation of NF-κB has been well-characterized in various human cancers including hepatocellular carcinoma (HCC) [9]–[10]. In the classical NF-κB signaling pathway, heterodimeric NF-κB (p50–p65) is arrested by inhibitors of the κB protein (IκB) in the cytoplasm. Upon stimulation of the NF-κB pathway, IκB is phosphorylated by the IKK complex, which is composed of a regulatory subunit NEMO (also known as IKKγ), and catalytic subunits IKKα, and IKKβ. The phosphorylated IκB is subsequently degraded and NF-κB (p50–p65) is translocated into the nucleus [7].

It is important to note that NF-κB is abnormally regulated by a number of human oncogenic viruses such as the human T cell leukemia virus type 1 [11], herpes virus [12], epstein-barr virus [13], and hepatitis B and C viruses [14]–[16]. The specific oncoproteins of these viruses target different sites of the NF-κB pathway, eventually leading to aberrant activation of NF-κB [16]. Among these viruses, hepatitis B virus (HBV) is associated with various liver diseases including liver inflammation, cirrhosis, and HCC [17]–[18]. The HBV X protein (HBx) is known to play a critical role in HBV-related HCC development through a number of mechanisms such as stimulation of cell growth-related transcription and cancer-related signaling pathways [19]–[20]. In particular, it is well-documented that HBx activates NF-κB signaling [21]–[27]. Moreover, several studies proposed involvement of several host protein partners in this event [28]–[32]. Recently, we showed that the abnormally over-expressed RPS3a in HBV-associated HCC tissues stabilizes the HBx protein by its novel chaperoning activity and enhances HBx-induced NF-κB signaling [31].

HBx is a multifunctional regulatory protein that exerts different functions depending on the cell type and cell conditions. These functions are suggested to be involved in HBV pathogenesis through the interaction of HBx with various cellular proteins. Previously, we reported that HBx interacts with c-FLIP_L_ and c-FLIP_S_ via their N-terminal DED1 domain and inhibits their recruitment to DISC [33]. In this study, we explored whether p22-FLIP interacts with HBx and modulates its function, especially in HBx- or p22-FLIP-mediated NF-κB activation. We show that p22-FLIP synergistically enhances HBx-induced NF-κB activation in liver cells. Additionally, p22-FLIP hyperactivates the HBx-induced NF-κB signal through the formation of a ternary complex among HBx, p22-FLIP, and NEMO. Thus, our present study demonstrates a novel mechanism for HBx-induced NF-κB activation and expands the understanding of how HBV affects the NF-κB system for HBV-associated liver diseases.

## Results

### Expression of Endogenous p22-FLIP in Human Hepatoma Cells

**Figure 1:**
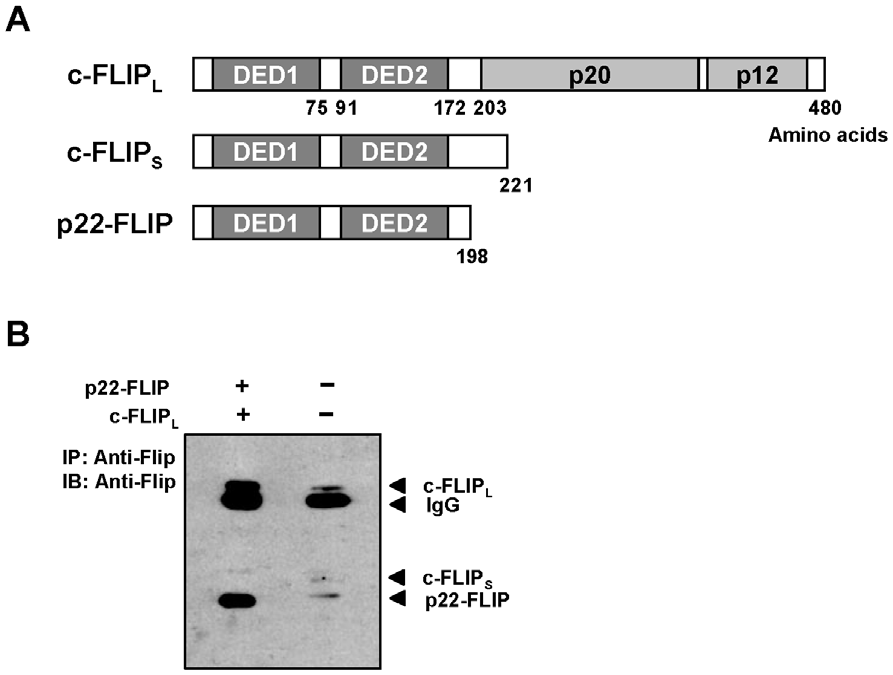
Detection of endogenous p22-FLIP in human liver cells. (**A**) Schematic illustrations of c-FLIP_L_, c-FLIP_S_, and p22-FLIP. (**B**) Detection of endogenous p22-FLIP in Huh7 cells. Approximately 9.5×10^6^ Huh7 cells were used for immunoprecipitation and Western blot. As positive controls, plasmids for p22-FLIP (1 µg) and c-FLIP_L_ (1 µg) were co-transfected in Huh7 cells and subjected to immunoprecipitation.

p22-FLIP was first identified in lymphocytes as a cleavage product of c-FLIP_L_ and c-FLIP_S_ by procaspase 8 [6]. Although we previously showed that c-FLIP_L_ and c-FLIP_S_ are expressed in liver cells [33], until now, the existence of p22-FLIP has not yet been identified in liver cells. Endogenous p22-FLIP shares the N-terminal domain (amino acids 1–198) with c-FLIP_L_ and c-FLIP_S_ as depicted in Figure 1A. Moreover, it is reported to be a strong inducer of NF-κB whereas c-FLIP_L_ has no function on NF-κB activation [6]. As NF-κB activation is demonstrated to be closely related with liver diseases and HBV-related HCC [17], we first investigated whether the p22-FLIP protein is present in Huh7 human hepatoma cells. Lysates of Huh7 cells were immunoprecipitated using anti-Flip antibody which recognizes the N-terminal domain of c-FLIP_L_. As a positive control, plasmid encoding p22-FLIP was cloned and co-transfected with c-FLIP_L_ in Huh7 cells. We were able to detect endogenous p22-FLIP (about 22 kDa) as well as c-FLIP_L_ and c-FLIP_S_ in non-transfected Huh7 cells (Figure 1B). The existence of p22-FLIP in liver cells was also observed in our previous report [33]. Even though p22-FLIP had not been discovered at that time, the p22-FLIP band was clearly detected (Figure 6A in ref. [33], lower band of c-FLIP_S_) in HepG2, another line of human hepatoma cells. In addition, p22-FLIP was detected in the last figure in present study. These results demonstrate that endogenous p22-FLIP is constitutively present in human hepatoma cells.

### p22-FLIP Synergistically Enhances HBx-induced NF-κB Activation

**Figure 2:**
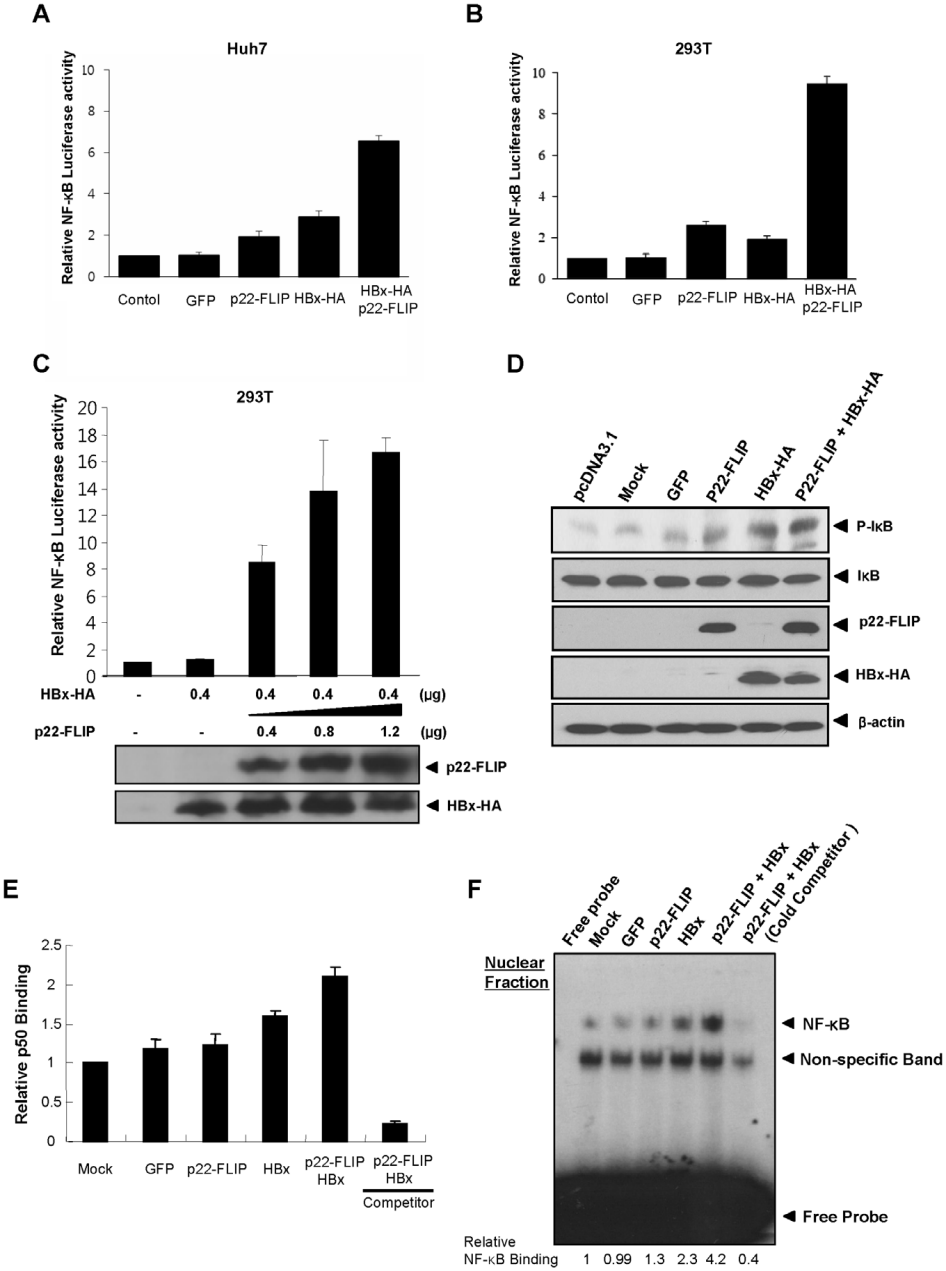
p22-FLIP synergistically up-regulates HBx-mediated NF-κB signaling. (**A–B**) Relative NF-κB activity after co-transfection of pNF-κB-Luc and p22-FLIP plasmid with/without HBx-HA plasmid in Huh7 and 293T cells, respectively. pEGFP were transfected for the monitoring of transfection efficiency and negative control. (**C**) Dose-dependent activation of HBx-mediated NF-κB by p22-FLIP. pNF-κB-Luc (0.25 µg) and HBx-HA plasmid (0.4 µg) were co-transfected with increasing amounts of p22-FLIP (0∼1.2 µg) in 293T cells. Total transfected DNA amounts were adjusted using the empty vector (pCMV). The expression levels of p22-FLIP and HBx were determined by Western blot. (**D**) The level of phospho-IκB (P-IκB) was determined by western blot. The plasmids of p22-FLIP (1 µg) and HBx-HA (1 µg) were co-transfected in Huh7 cells. After 48 hours, the levels of P-IκB and total IκB were analyzed by western blot. (**E**) NF-κB ELISA was measured by p50 ELISA using nuclear extracts. The interaction of plate-bound NF-κB consensus DNA oligomer and NF-κB subunit (p50) in nuclear extracts was measured by chemiluminescence. (**F**) NF-κB electrophoretic mobility shift assay (EMSA). The [P^32^]-labeled NF-κB consensus DNA oligomer probe was reacted with nuclear extracts (3 µg) *in vitro*. Non-labeled NF-κB consensus oligomer (30 fold) was used for cold competition. Relative binding affinity was calculated by densitometry.

HBx has been implicated to play an important role in HBV pathogenesis, especially in HBV-related HCC [19], [20]. Since the HBx protein is well established to induce activation of NF-κB in liver cells [19]–[27], we questioned whether cellular p22-FLIP would affect HBx-induced NF-κB activity. NF-κB activity was measured using the NF-κB luciferase reporter system after transfection of HBx, p22-FLIP, and both plasmids. As shown in previous reports, p22-FLIP [6] and HBx [21]–[27] individually enhanced NF-κB signaling, although their enhancing effects on NF-κB activation were marginal (Figure 2A and 2B). However, co-expression of p22-FLIP with HBx strongly enhanced NF-κB activation in Huh7 cells thereby exerting a synergistic effect as opposed to individual expression. This observation was more prominent in 293 T cells (Figure 2B). To further confirm the effect of p22-FLIP on HBx-related NF-κB activation, NF-κB activity was measured after dose-dependent transfection of p22-FLIP. As shown in Figure 2C, HBx-induced NF-κB activity was greatly enhanced in proportion to the dose of p22-FLIP. Next, we examined the level of phosphorylated IκB which is related to NF-κB activation. The result showed that co-expression of p22-FLIP and HBx enhances the phosphorylation of IκB (Figure 2D).

Finally, to verify the enhancing effect of p22-FLIP on HBx-induced NF-κB, we employed other experimental methods, including NF-κB ELISA and electrophoretic mobility shift assay (EMSA). Because activated NF-κB (p50–p65 heterodimer) translocates into the nucleus in the classical NF-κB signaling pathway, we speculated that the measurements of NF-κB level in the nucleus might be direct evidence of NF-κB activation. Therefore, we determined the translocated level of p50 by chemiluminescence after capturing the complex of plate-bound NF-κB consensus DNA oligomer and p50 (Figure 2E). The level of nuclear p50 was significantly increased by p22-FLIP and HBx, and this was further confirmed by NF-κB EMSA using a nuclear extract (Figure 2F). EMSA also showed that NF-κB is significantly activated by coexpression of p22-FLIP and HBx.

Taken together, our data demonstrate that p22-FLIP synergistically activates HBx-induced NF-κB signaling, suggesting its potential role in HBV-related HCC development.

### Knock-down of p22-FLIP Abolished HBx-induced NF-κB Activation

**Figure 3:**
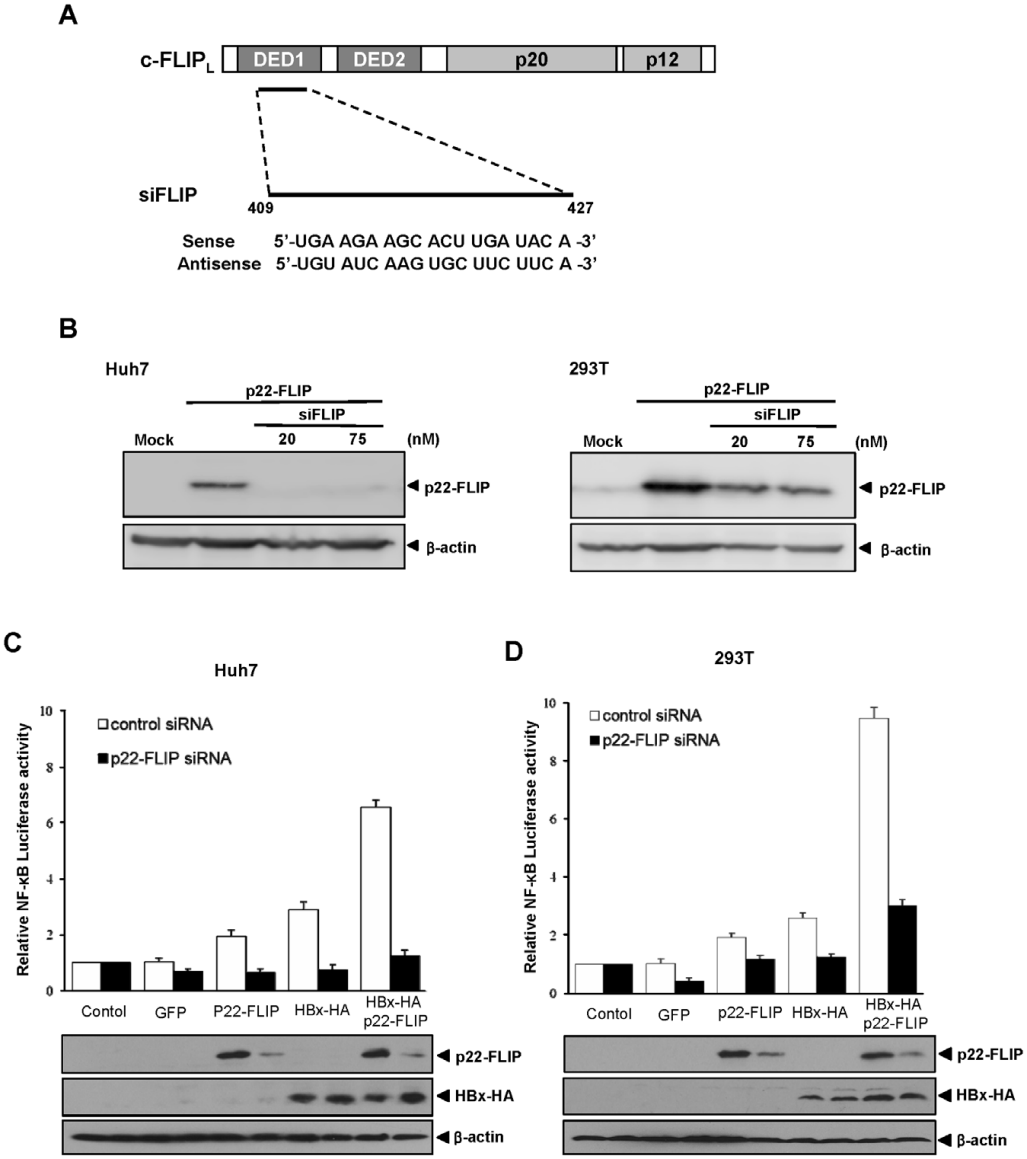
The treatment of siFLIP abolished the synergistic effect of p22-FLIP on HBx-mediated NF-κB activation. (**A**) A schematic representation of the siFLIP design. (**B**) The knock-down effect of siFLIP on p22-FLIP. The plasmid for p22-FLIP was co-transfected with siFLIP in Huh7 (left panel) and 293T cells (right panel). At 48 hours post-transfection, the expression level of p22-FLIP was analyzed by Western blot. (**C–D**) The effect of p22-FLIP knock-down on NF-κB activity. The indicated plasmids and pNF-κB-Luc (0.25 µg) were co-transfected with siFLIP (20 nM) or control siRNA in Huh7 cells(C) and 293T cells (D), respectively. Relative NF-κB activity was determined as described above. The expression levels of p22-FLIP and HBx were analyzed by western blot.

To confirm the synergistic effect of p22-FLIP on HBx-induced NF-κB activation, knock-down studies of p22-FLIP were performed after synthesis of siRNA against p22-FLIP. siFLIP was designed to recognize the DED1 domain of p22-FLIP and c-FLIP_L_ (Figure 3A). We verified the effect of siFLIP by degradation of p22-FLIP after over-expression in Huh7 and 293T cells (Figure 3B). Following this, we performed NF-κB luciferase assay after knock-down of p22-FLIP. The expression level of HBx was not affected by siFLIP treatment (Figure 3C and 3D). However, treatment of siFLIP completely abolished the synergistic effect of p22-FLIP on HBx-induced NF-κB activation in Huh7 and 293T cells, whereas the control siRNA showed no effect (Figure 3C and 3D). On a side note, treatment of siFLIP in cells expressing both p22-FLIP and HBx dramatically abolished the effect of p22-FLIP on HBx-induced NF-κB activation down to the basal level (control) in Huh7 cells (Figure 3C).

**Figure 4:**
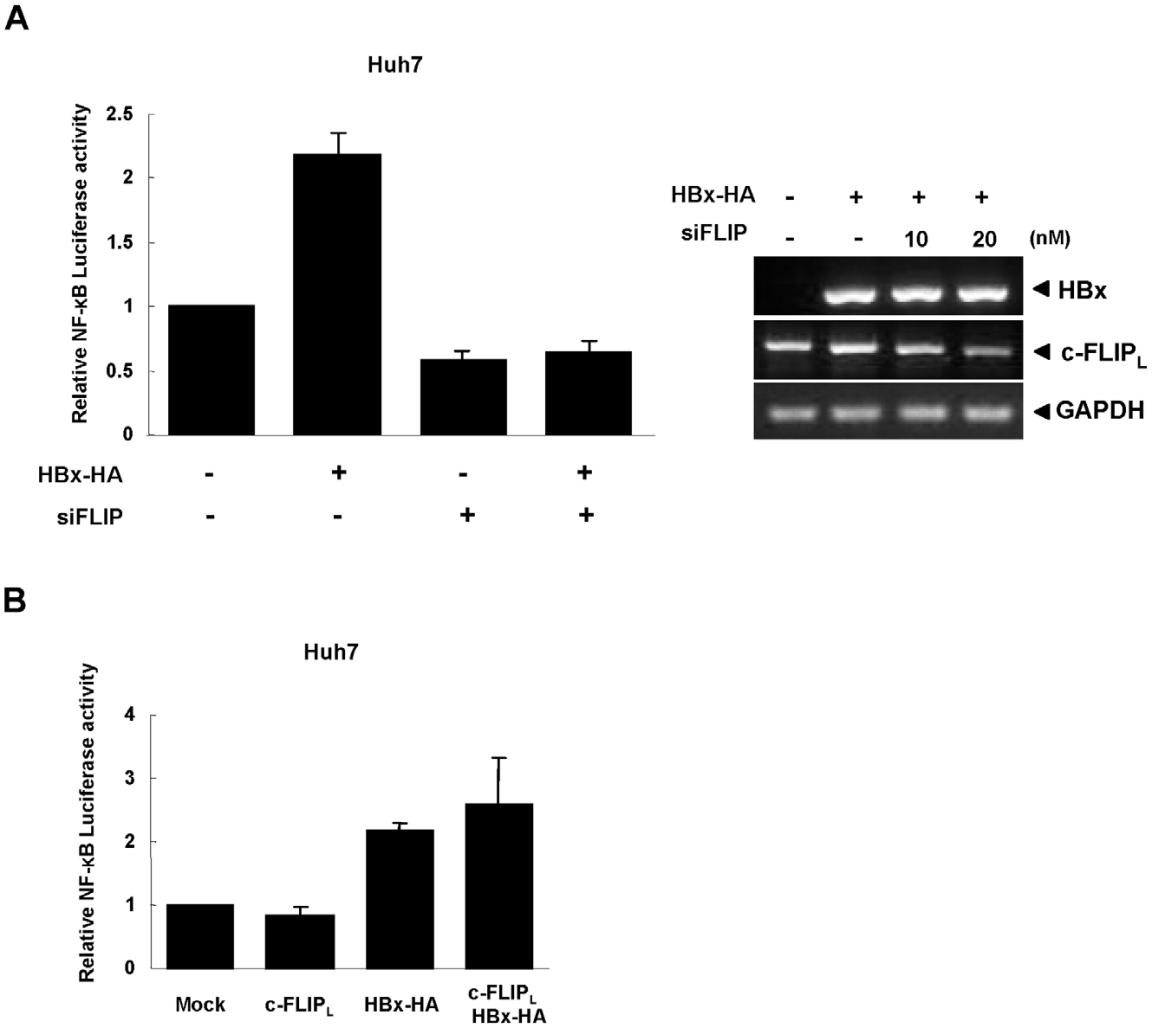
Endogenous p22-FLIP is involved in HBx-mediated NF-κB signal. (**A**) The effect of endogenous p22-FLIP on NF-κB activation. NF-κB activity was determined after co-transfection with pNF-κB-Luc and siFLIP (20 nM) with/without HBx-HA in Huh7 cells (left panel). The levels of mRNAs after treatment of siFLIP (right panel). HBx-HA was co-transfected with siFLIP with the indicated concentration. The expression levels were analyzed by semi-qRT-PCR using specific primers, respectively. GAPDH was used as a loading control. (B) The effect of c-FLIP_L_ expression on HBx-mediated NF-κB activation. NF-κB activity was measured after co-transfection of pNF-κB-Luc and the indicated plasmids in Huh7 cells.

In addition, HBx-induced NF-κB activation was significantly decreased by siFLIP treatment in both Huh7 and 293T cells (HBx-HA in Figure 3C). Since those cells were not transfected with p22-FLIP, these results imply that endogenous (basal level) p22-FLIP might play some role in the process of HBx-induced NF-κB activation. To confirm the involvement of endogenous p22-FLIP in HBx-induced NF-κB activation, we measured HBx-induced NF-κB activity after treatment of siFLIP without transfection of p22-FLIP. Under this condition, NF-κB luciferase activity was significantly reduced to less than the basal level (Figure 4A, left), implying that endogenous p22-FLIP is involved in HBx-induced NF-κB signaling. Notably, the knock-down of c-FLIP leading to downregulation of p22-FLIP reduced the basal level of NF-κB activity, suggesting that endogenous (basal level) p22-FLIP plays a role in the maintenance of basal NF-κB activity in unstimulated liver cells. In this condition, treatment of siFLIP reduced the expression of c-FLIP while the expression level of HBx was not influenced (Figure 4A, right panel).

The c-FLIP family share the common N-terminal DED1 domain as shown in Figure 1A, and p22-FLIP is generated by cellular processing of c-FLIP_L_ [6]. Therefore, treatment of siFLIP can knock-down the expression of both c-FLIP_L_ and p22-FLIP proteins. To exclude the possibility that the reduction of NF-κB activity by siFLIP is caused by the knock-down of c-FLIP_L_ in our system, we checked the effect of c-FLIP_L_ expression on NF-κB activation and HBx-induced NF-κB activation (Figure 4B). However, we found that over-expression of c-FLIP_L_ had no effect on both basal NF-κB and HBx-induced NF-κB activation in Huh7 cells, suggesting that the c-FLIP effect on NF-κB activation is solely due to the p22-FLIP protein.

Overall, our results demonstrate that both basal NF-κB and the synergistic effect of HBx-induced NF-κB activation are wholly attributed to p22-FLIP, not c-FLIP_L_.

### HBx Potentially Forms a Ternary Complex with p22-FLIP and NEMO

**Figure 5:**
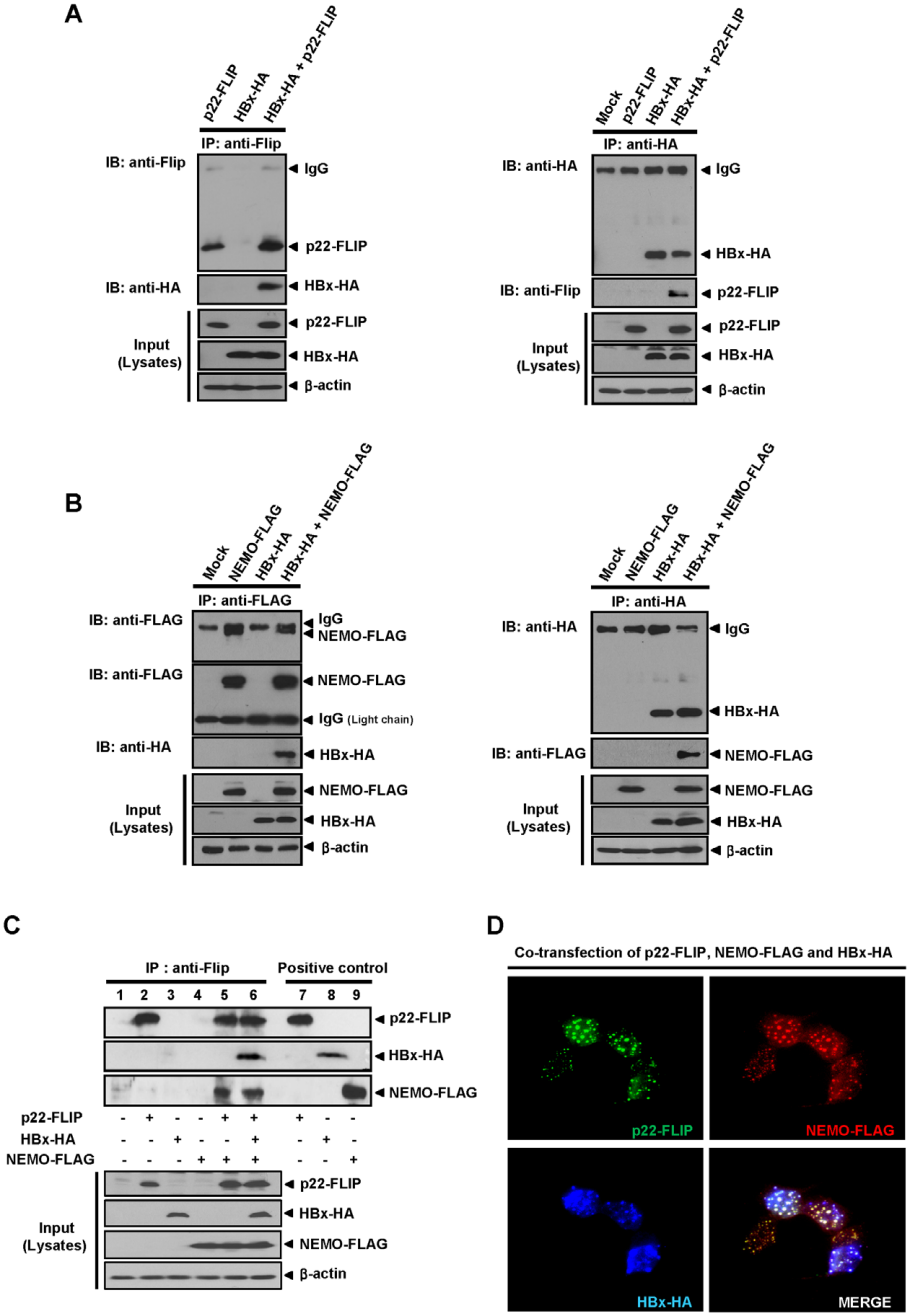
HBx, p22-FLIP and NEMO potentially form a ternary complex. (**A**) Interaction of HBx with p22-FLIP in Huh7 cells. After co-transfection of HBx-HA and p22-FLIP plasmids in Huh7 cells (approximately 6×10^5^), cell lysates were immunoprecipitated by anti-Flip (left panel) or anti-HA (right panel) antibodies, respectively. (**B**) HBx physically interacts with NEMO in Huh7 cells. HBx-HA and NEMO-FLAG plasmids were co-transfected in Huh7 cells as indicated. After 48 hours, cell lysates were immunoprecipitated using anti-FLAG (left panel) or anti-HA (right panel), respectively. Binding of HBx-HA and NEMO-FLAG proteins was examined by Western blot using anti-HA (left panel) or anti-FLAG (right panel), respectively. To separate the bands for NEMO-FLAG and IgG, the secondary antibody specific for light chain (Fab) was used. (**C**) The ternary complex formation among HBx, p22-FLIP, and NEMO. At 48 hours post-transfection of p22-FLIP, HBx-HA and NEMO-FLAG plasmids, cell lysates were immunoprecipitated with anti-Flip antibody. The precipitated immune complex was analyzed by Western blot analysis using the indicated antibodies (anti-Flip, anti-HA and anti-FLAG), respectively. Cell lysates were used as a positive control for the expression of each protein (lane 7∼9). (**D**) Co-localization assay of HBx, p22-FLIP, and NEMO in liver cells. At 24 hours post-transfection of HBx, p22-FLIP, and NEMO, immunofluorescence assay was performed using each antibody (anti-HA, anti-Flip, and anti-FLAG). After binding with the fluorescence labeled second antibodies, the stained cells were visualized by fluorescence microscopy (magnification, ×400). The white color represents co-localization of three proteins.

We next investigated the underlying molecular mechanism of how p22-FLIP exerts the synergistic effect on HBx-induced NF-κB activation. It has been previously reported that p22-FLIP interacts with NEMO, a member of the IKK complex in immune cells [6]. Our previous report has shown that HBx interacts with c-FLIP_L_ via its N-terminal DED1 domain [33]. Therefore, we hypothesized that HBx might bind to the p22-FLIP-IKK complex through the DED1 domain of p22-FLIP and may form a ternary complex which can continuously hyperactivate NF-κB signaling. To prove this, we first performed co-immunoprecipitation (CoIP) assay after cotransfection of p22-FLIP and HBx in Huh7 cells, and investigated whether HBx binds to p22-FLIP. CoIP data using anti-Flip and anti-HA antibodies clearly showed that HBx interacts with p22-FLIP (Figure 5A). After this, we examined whether HBx interacts with NEMO by using CoIP, and found that HBx is also associated with NEMO (Figure 5B). The interaction between HBx and NEMO was verified by CoIP using anti-HA antibody (right panel). These data revealed that HBx interacts with both p22-FLIP and NEMO.

**Figure 6:**
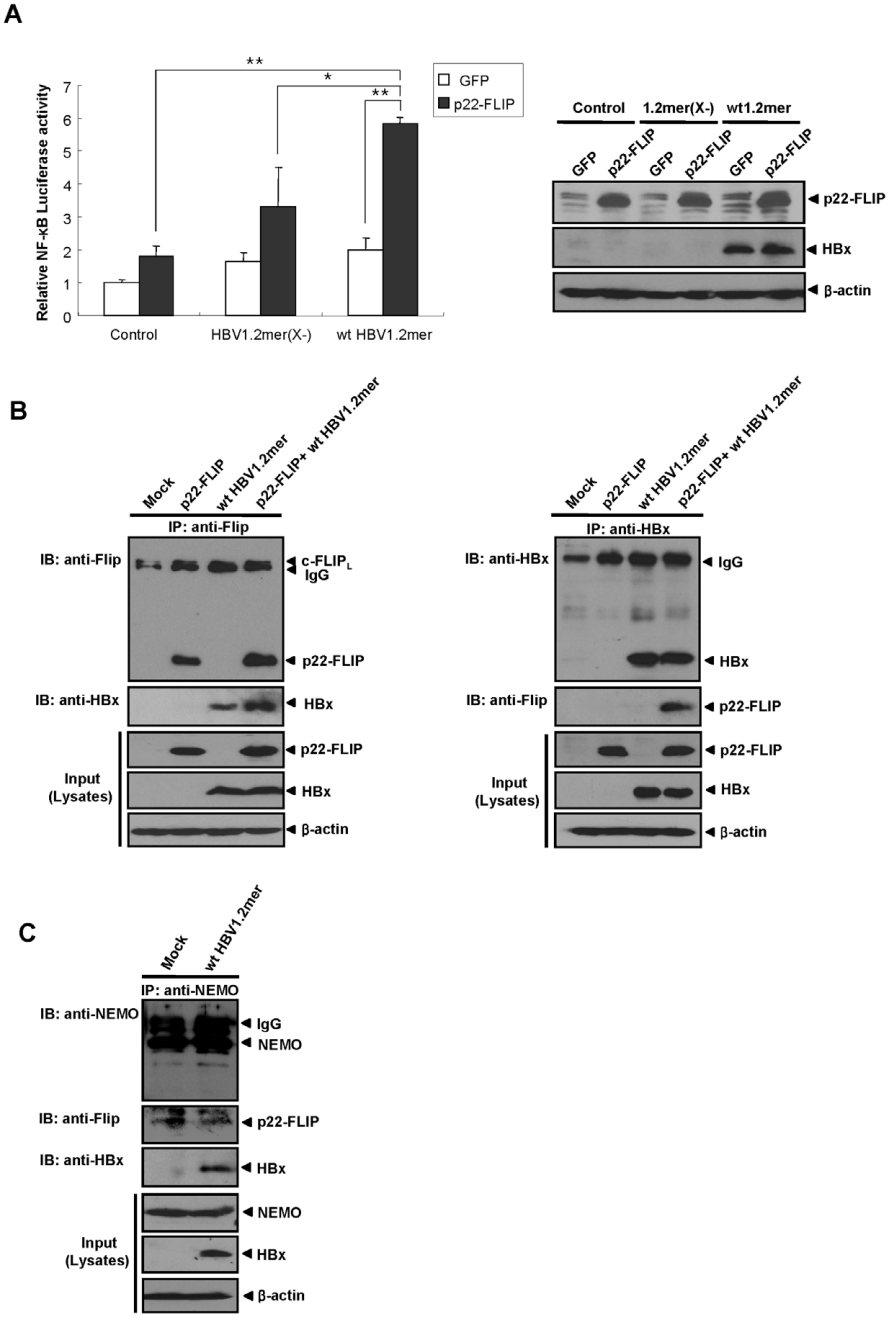
p22-FLIP synergistically enhances HBx-induced NF-κB signaling in the context of replication-competent HBV through interaction with HBx. (A) p22-FLIP synergistically enhances HBx-mediated NF-κB signaling in the context of HBV full genome. Relative NF-κB activity (left panel) was measured at 48 hours post-transfection of pNF-κB-Luc(0.25μg) and the indicated plasmids (0.4μg) in Huh7 cells. Total amounts of DNA were adjusted by pCMV vector. Results were obtained by at least four independent experiments (*, P = 0.011; **, P<0.001). Expression levels of p22-FLIP and HBV genome-driven HBx were determined by Western blot analysis using the indicated antibodies (right panel). (B) p22-FLIP physically interacts with HBV genome-driven HBx in liver cells. At 72 hours post-transfection of wt HBV1.2mer (2μg) and p22-FLIP (2μg) plasmids, Huh7 cell lysates were immunoprecipitated with anti-Flip (left panel) or anti-HBx (right panel) antibodies, respectively. Total amounts of transfected DNA were adjusted by pcDNA3.1 vector. Western blot analysis was carried out using the indicated antibodies. Cell lysates were used as a positive control. (C) Potential formation of ternary complex among endogenous p22-FLIP, NEMO, and genome-driven HBx in Huh7 cells. Approximately 7.8×10^6^ Huh7 cells were transfected with wt HBV1.2mer plasmids and cultured for 72 hours. Thereafter, cell lysates were immunoprecipitated using anti-NEMO antibody and blotted by anti-NEMO, anti-Flip, and anti-HBx antibodies.

To determine whether HBx forms a ternary complex with p22-FLIP and NEMO, we performed CoIP assay after cotransfection of HBx, p22-FLIP and NEMO in Huh7 cells. Expression of three proteins were verified (Figure 5C, Input) and used as positive controls (Figure 5C, 7∼9 lanes). When p22-FLIP was immunoprecipitated using anti-Flip antibody, both HBx and NEMO were precipitated together with p22-FLIP, suggesting that a ternary complex is potentially formed by HBx, p22-FLIP and NEMO (Figure 5C, lane 6). It is likely that HBx associates with the p22-FLIP-IKK complex and forms a ternary complex, which can hyperactive the downstream signaling of NF-κB. According to above data (Figure 3C, 3D, 4A), NF-κB signal was dramatically decreased when the overexpressed or endogenous p22-FLIP, a component of the ternary complex, was knocked-down. These data suggest that potential formation of a ternary complex is necessary for HBx-induced NF-κB activation.

Finally, to confirm formation of a ternary complex among HBx, p22-FLIP and NEMO, co-localization assay was performed (Figure 5D). As expected, the data clearly showed that three proteins were co-localized in Huh7 cells.

### p22-FLIP Synergistically Enhances HBx-induced NF-κB Signaling through Forming a Ternary Complex in the Context of Replication-competent HBV

Finally, to confirm whether the mutual synergistic effect between p22-FLIP and HBx on NF-κB activation can occur during the natural course of HBV infection, we investigated HBx-induced NF-κB activity using a replication-competent wild type HBV full genome (wt HBV1.2mer). wt HBV1.2mer is assumed to express the physiological level of the HBx protein and also other viral proteins such as polymerase, surface, and core through its own viral promoter. An HBx-deficient HBV genome (HBV1.2mer(X-)) was used as a negative control [33], [34]. The expression level of genome-driven HBx was not changed by the expression of p22-FLIP and *vice versa* (Figure 6A, right panel). NF-κB luciferase assay showed that p22-FLIP synergistically enhanced HBV-induced NF-κB signaling, whereas it only showed an additive effect on NF-κB activity when the HBx-deficient HBV genome was transfected (HBV 1.2mer(X-)) (Figure 6A, left panel). This result demonstrates that the enhanced NF-κB signal is also HBx-dependent in the context of the full HBV genome, which is consistent with the previous data shown in the HBx over-expression system (Figure 2).

To further confirm whether the enhancement of NF-κB signaling by p22-FLIP is also mediated by the interaction with the genome-driven HBx, we performed CoIP assay using anti-Flip or anti-HBx antibodies in Huh7 cells. The result clearly showed that p22-FLIP interacts with the genome-driven HBx (Figure 6B). The genome-driven HBx was shown to interact with both p22-FLIP and endogenous c-FLIP_L_ as reported in previous study [33]. Notably, the amount of bound HBx to the lane of p22-FLIP overexpression was significantly increased, implying that p22-FLIP associates with the genome-driven HBx (Figure 6B left).

Finally, we investigated whether the ternary complex is formed by the endogenous p22-FLIP, NEMO, and genome-driven HBx in physiological condition. The result of CoIP using NEMO antibody revealed that the endogenous p22-FLIP and NEMO potentially form a ternary complex with the genome-driven HBx (Figure 6C).

Taken together, the results collectively suggest that the synergistic effect of p22-FLIP on HBx-induced NF-κB activation through forming a ternary complex might occur during the natural course of HBV infection.

## Discussion

Chronic HBV infection is closely associated with the development of HCC, in which HBx has been reported to play critical roles [17]. HBx is a multi-functional viral protein and is thought to influence the physiological balance of cells in a number of ways, including control of cell cycle, induction of proto-oncogenes, and transactivation of key genes related to cell growth [17]–[20]. Moreover, HBx was reported to activate NF-κB signaling through modulation of cellular signal pathways [21]–[27]; HBx activates NF-κB by inhibiting both IκB and NF-κB1 precursor, known as p105 [24], by activating Ras–Raf–MAPK pathways [26] and oxidative stress [27]. Moreover, NF-κB activation by HBx is associated with a variety of cellular proteins [28]–[32]. The association between HBx and oncogene AIB1 in the activation of NF-κB signal transduction and cell invasiveness is reported [32], [35]. The chaperoning function of RPS3a is involved in HBx-mediated NF-κB activation [31]. NF-κB activation by HBx regulates a number of genes that control cell growth and tumorigenesis [8], [9]. Therefore, it is assumed that NF-κB activation by HBx is one of the most important factors for HBV-related HCC development. In this study, we found another cellular target, p22-FLIP, which potentially forms a ternary complex with HBx and NEMO, for the process of HBx-mediated activation of NF-κB.

A number of accumulating reports have clearly elucidated that c-FLIP_L_ inhibits death receptor-mediated apoptosis by blocking the recruitment of procaspase 8 into DISC [1]–[4]. Along with the anti-apoptotic function, c-FLIP_L_ has also been reported to induce NF-κB activation and to be involved in lymphocyte proliferation [36]–[40]. However, c-FLIP_L_ did not affect NF-κB activity in liver cells in our system (Figure 4B). The underlying molecular mechanism of NF-κB activation by c-FLIP_L_ regulation has not been clearly elucidated. In human herpes virus 8 (HHV-8), viral FLIP (also known as K13) induces the NF-κB activation through association with IKK complex [41]. Recently, p22-FLIP was characterized as a cleavage product of c-FLIP_L_ and an inducer of NF-κB activation in lymphocyte and dendritic cells (DCs) through forming a complex with NEMO, a member of the IKK complex [6]. Therefore, one possible explanation could be that p22-FLIP is an executing molecule for c-FLIP_L_-mediated NF-κB activation. The cell type-specific activation of c-FLIP_L_-mediated NF-κB activation is probably dependent at the cellular level of p22-FLIP.

Previously, we found that HBx sensitizes hepatocytes to death-inducing signals through interaction with c-FLIP_L_ [33]. In this study, we demonstrated that HBx activates NF-κB through interaction with p22-FLIP. These somewhat controversial results suggest that HBx can control (or switch) the fate of cells toward apoptosis or survival by the cellular levels of c-FLIP_L_ and p22-FLIP. Therefore, these data stress the importance of stimulating signal(s) that participate in the conversion of c-FLIP_L_ into p22-FLIP. Further investigations are warranted to understand the fate of cells which are infected with HBV.

In the present study, we first identified the endogenous expression of p22-FLIP in hepatoma cells (Figure 1B), and suggested the role of p22-FLIP for NF-κB activation in liver cells which are similar to immune cells. Contrary to our expectations, the effect of p22-FLIP on NF-κB activation was marginal (Figure 2). It is likely that NF-κB activation by p22-FLIP might be suppressed by unknown regulatory mechanisms in liver cells. However, NF-κB activation was synergistically hyperactivated under co-expression of p22-FLIP with HBx (Figure 2 and 6A) through the potential formation of a ternary complex among HBx, p22-FLIP and NEMO in hepatoma cells (Figure 5C, 5D, and 6C). There is the possibility that HBx, p22-FLIP and NEMO form separate binary complexes instead of ternary complex. To form the separate binary complexes, the binding site in p22-FLIP need to be shared (or overlapped) by HBx and NEMO. Therefore, there would be competitive interaction between NEMO and HBx with p22-FLIP. However, as shown in Figure 6C, the amount of bound p22-FLIP to NEMO was not significantly changed by overexpression of HBx (wt HBV1.2mer), suggesting that there is no competitive interaction between NEMO and HBx with p22-FLIP. Similarly, the amounts of bound NEMO to p22-FLIP were not changed by overexpression of HBx (Figure 5C). Data suggest that HBx, p22-FLIP, and NEMO are likely to form a ternary complex rather than separate binary complexes. Knock-down studies also revealed that endogenous p22-FLIP is involved in HBx-induced NF-κB activation (Figure 3 and 4). Collectively, it is clear that infection of HBV stimulates c-FLIP_L_- or p22-FLIP-mediated NF-κB signals in liver cells by interacting with HBx, which might be involved in the pathogenesis of HBV.

Over-expression of c-FLIP_L_ is observed in most cancers including hepatocellular carcinoma [42]–[47]. It would be worth investigating whether p22-FLIP is also over-produced in many types of cancer tissues because the over-expressed c-FLIP_L_ might be converted to p22-FLIP through cleavage by procaspase 8 during cancer development. It is worth mentioning that human HCCs have been shown to be resistant to death receptor-mediated apoptosis, and c-FLIP was detected in 83% of human HCC tissues whereas it was absent in normal hepatic tissues [47]. In this regard, it will be of interest to investigate whether or how HBV infection affects the conversion of c-FLIP_L_ to p22-FLIP which might be involved in HCC development.

Synergistic activation of NF-κB through a ternary complex is demonstrated in this study. This finding is important in the aspect of viral escape from host immune system. One of the major mechanisms for HBV clearance is the cytotoxic T lymphocyte (CTL)-mediated induction of interferon gamma, tumor necrosis factor alpha, and interleukin 2 [48]. Therefore, hepatocytes infected with HBV are under pressure of clearance. The induction of p22-FLIP mediated NF-κB signal which is essential for cell survival and proliferation might give hepatocytes an opportunity to evade host immune pressures and eventually lead to persistent infection.

In conclusion, we showed that HBx potentially forms a ternary complex with p22-FLIP and NEMO, and regulates NF-κB signals. Our findings will extend the understanding of HBx-induced NF-κB activation and pathophysiology of virus-mediated liver diseases.

## Materials and Methods

### Plasmid Construction and Reagents

The details of used plasmids for HBx-HA (subtype *ayw*) and c-FLIP_L_ are described in our previous reports, respectively [31], [33]. Plasmids for the replication-competent HBV genome (wt HBV1.2mer and HBV1.2mer(X-)) [33], [34] and NEMO-FLAG [49] were kindly provided by Prof. WS Ryu (Yonsei Univ.) and Prof. TH Lee (Yonsei Univ.), respectively. An expression plasmid for p22-FLIP was amplified by PCR using c-FLIP_ L_ plasmid as template and subcloned into the pcDNA3.1 vector (Invitrogen) using EcoRI and XbaI. Antibodies (Dave-2 and NF6) for the detection of c-FLIP_ L_ and p22-FLIP were purchased from Alexis Biochemicals (San Diego, CA). To detect genome-driven HBx proteins, polyclonal anti-HBx antibody (MYBiosource, CA, USA or Biovendor, Modrice, Czech Republic) was used. The antibodies for NEMO, phospho-IκB and IκB were obtained from Santa Cruz Biotechnology (Santa Cruz, CA, USA). Antibodies for FLAG and HA-tagged proteins, β-actin, and secondary antibodies were obtained from Sigma (St. Louis, MO). For immunocytochemistry, Alexa Fluor-568, Alexa Fluor-350, and Alexa Fluor-488 (FITC) antibodies were purchased from Invitrogen (Carlsbad, CA).

### Cell Culture and Transfection

Huh7 and 293T cell lines were obtained from the American Type Culture Collection (ATCC, Manassas, VA). Both cell lines were maintained in DMEM supplemented with 10% heat-inactivated FBS, 1% penicillin, and 1% streptomycin (Gibco BRL, Oregon, USA). Transient transfection or co-transfection were carried out using Lipofectamine 2000 (Invitrogen, Carlsbad, CA) or LT1 (Mirus, Madison, WI) according to the manufacturer’s instructions, respectively at 60–80% cell confluency.

### Western Blot Analysis and Immunoprecipitation Assay

Western blot analysis and immunoprecipitation assays were performed as described previously [31]. Briefly, after 48 hours transfection, cells were lysed with lysis buffer [50 mM Tris-HCl pH 8.0, 10 mM NaCl, 0.5% NP-40, protease inhibitor cocktail (Sigma, St.Louis, MO)]. For immunoprecipitation, the clarified cell lysates were incubated with primary antibodies with gentle rotation at 4°C after precleaning using protein-A agarose (or protein-G agarose for rat IgG) (Roche, Mannheim, Germany) for 3 hours. After overnight, lysates and antibody mixture were treated with 10 µl of protein-A (or G agarose), followed by 2 additional hours of incubation under the same conditions. After 3 times of washing with PBS, the immune-complex was mixed with 10 µl of 2× SDS sample buffer, and then, SDS-PAGE and western blot were performed.

### Luciferase Assay for Detection of NF-κB Activation

Approximately 10×10^4^ 293T cells (or 4×10^4^ Huh7 cells) were seeded on 12-well culture plates. After 1 day, cells were transiently transfected with DNA mixtures (GFP, p22-FLIP or c-FLIP_L_, HBx-HA plasmids) including 0.5 µg NF-κB luciferase (pNF-κB-Luc, Stratagene) and 0.2 µg β-gal reporter gene. Empty vector was used for adjusting the total amount of transfection DNA. At 16 hours post-transfection, cells were lysed by lysis buffer (Promega, Madison, WI), and lysates were assayed for NF-κB luciferase activity using Luciferase Assay System (Promega, Madison, WI). Each raw data were normalized by the results of β-gal assay. Data were collected from the results of at least 3 independent experiments.

### RNA Interference and Semi-quantitative RT-PCR

For RNA interference of p22-FLIP, siRNA against N-terminal sequences of c-FLIP_L_ was synthesized by Samchully Pharm (Seoul, Korea) as follows: Sense, 5′-UGA AGA AGC ACU UGA UAC A -3′ and antisense, 5′-UGU AUC AAG UGC UUC UUC A-3′. Transfection of each annealed siRNA (20 nM) was performed using Lipofectamine 2000 (Invitrogen, Carlsbad, CA).

To verify the expression level of target mRNA, semi-quantitative RT-PCR was carried out. After isolation of total RNA using Trizol solution (Invitrogen, Carlsbad, CA), the total RNA(2 µg) was mixed with oligo dT primer and reverse transcriptase (Intron Biotechnology, Seoul, Korea) for cDNA systhesis. Finally, PCR was performed using a PCR mixture [1 µl cDNA product, 0.5 U ex*Taq* polymerase (Takara, Shiga, Japan), 0.25 mM dNTP and 0.5 mM target specific primers (c-FLIP_L_ or HBx or GAPDH)]. The specific primers are as follows: c-FLIP_L_ sense, 5′-ATG TCT GCT GAA GTC ATC C -3′ and antisense, 5′-TGC TGG GAT TCC ATA TG-3′; HBx sense 5′-AAA AAG TTG CAT GGT GCT GGT GAA C-3′ and antisense, 5′-GCG CGG GAC GTA CTT TGT-3′; GAPDH sense, 5′-CGT CTT CAC CAC CAT GGA GA-3′ and antisense, 5′-CGG CCA TCA CGC CAC AGT TT-3′.

### NF-κB Electrophoretic Mobility Shift Assay (EMSA) and NF-κB ELISA

The details of NF-κB electrophoretic mobility shift assay (EMSA) and EMSA-ELISA are described in our previous report [31]. In brief, after separation of the nuclear fraction from Huh7 cells, the nuclear extracts were used for NF-κB EMSA and NF-κB ELISA (EZ-Detect Transcription Factor Kits for NF-κB p50, Pierce, Rockford, IL). For EMSA, after preincubation of the nuclear extracts (3 µg) with poly(dI-dC) in reaction buffer [10 mM Tris-Cl(pH7.5), 100 mM Nacl, 1 mM EDTA, 0.5 mM DTT, 10% glycerol], the [^32^P]-labeled NF-κB consensus oligonucleotide (Promega, Madison, WI) was treated. A complex formed by the probe DNA and protein (activated NF-κB) was subjected to electrophoresis. After gel drying, autoradiography was obtained to identify the DNA-protein complex. Unlabeled NF-κB oligonucleotide was treated as cold competitor before addition of the [^32^P]-labeled probe. For NF-κB ELISA analysis, the nuclear extracts (4 µg) were incubated with the supplied plate and antibodies according to the manufacture’s protocol. The level of p50 was measured by ELISA. The data represent the results of 5 independent experiments.

### Immunofluorescence and Co-localization Assay

Approximately 1.2×10^4^ Huh7 cells were plated on cover glass in 6 well plates. At 24 hours post-transfection with p22-FLIP, NEMO-FLAG and HBx, cells were fixed with 4% paraformaldehyde and permeabilized with 0.25% Triton X-100 for 5 min at room temperature. To reduce non-specific interaction, the cells were blocked with 10% BSA/PBS at 37°C for 30 min, and were incubated with primary antibodies (1∶400 with 3% BSA) for 1 hour at 37°C. After 3 times of PBS washing, the cells were treated with the following secondary antibodies (1∶500 with 3% BSA) for 1 hour at 37°C: NEMO-FLAG, anti-mouse IgG conjugated with Alexa 568; p22-FLIP, anti-rat IgG conjugated with Alexa 488 (FITC); HBx-HA, anti-rabbit IgG conjugated with Alexa 350. Immunofluorescence-labeled cells were mounted and visualized under a fluorescence microscope (Olympus, PLACE), equipped with an image analysis system (MetaMorph) (magnification, ×400).
